# Ablation of BRaf Impairs Neuronal Differentiation in the Postnatal Hippocampus and Cerebellum

**DOI:** 10.1371/journal.pone.0058259

**Published:** 2013-03-07

**Authors:** Verena Pfeiffer, Rudolf Götz, Chaomei Xiang, Guadelupe Camarero, Attila Braun, Yina Zhang, Robert Blum, Helmut Heinsen, Bernhard Nieswandt, Ulf R. Rapp

**Affiliations:** 1 Institute for Medical Radiation and Cell Research (MSZ), University of Würzburg, Würzburg, Germany; 2 Institute for Clinical Neurobiology, University Hospital Würzburg, Würzburg, Germany; 3 Rudolf Virchow Centre, DFG Research Centre for Experimental Biomedicine, University Hospital Würzburg, Würzburg, Germany; 4 Department of Psychiatry, Morphological Brain Research Unit, University of Würzburg, Würzburg, Germany; 5 Department of Molecular Mechanisms in Lung Cancer, Max Planck Institute for Heart and Lung Research, Bad Nauheim, Germany; Universidade Federal do ABC, Brazil

## Abstract

This study focuses on the role of the kinase BRaf in postnatal brain development. Mice expressing truncated, non-functional *BRaf* in neural stem cell-derived brain tissue demonstrate alterations in the cerebellum, with decreased sizes and fuzzy borders of the glomeruli in the granule cell layer. In addition we observed reduced numbers and misplaced ectopic Purkinje cells that showed an altered structure of their dendritic arborizations in the hippocampus, while the overall cornus ammonis architecture appeared to be unchanged. In male mice lacking *BRaf* in the hippocampus the size of the granule cell layer was normal at postnatal day 12 (P12) but diminished at P21, as compared to control littermates. This defect was caused by a reduced ability of dentate gyrus progenitor cells to differentiate into NeuN positive granule cell neurons. In vitro cell culture of P0/P1 hippocampal cells revealed that *BRaf* deficient cells were impaired in their ability to form microtubule-associated protein 2 positive neurons. Together with the alterations in behaviour, such as autoaggression and loss of balance fitness, these observations indicate that in the absence of BRaf all neuronal cellular structures develop, but neuronal circuits in the cerebellum and hippocampus are partially disturbed besides impaired neuronal generation in both structures.

## Introduction

Binding of growth factors to their cognate receptors leads to the activation of the RAS-RAF-MEK-ERK mitogen activated protein kinase (MAPK) cascade and is involved in regulation of many aspects of cellular growth and differentiation [1], [2]. The pathway consists of the small guanine nucleotide binding protein RAS and the protein kinases RAF, MEK, and ERK [1]. The activation of members of the RAF serine/threonine protein kinase family is initiated by RAS-GTP association with the RAS binding domain of RAF located at the N-terminus [3]. Most of the RAF functions appear to be mediated by phosphorylation/activation of the mitogen-activated protein kinase (MAPK)-extracellular signal-regulated kinases 1 and 2 (MEK1 and MEK2). ERK1 and ERK2 (extracellular-signal regulated protein kinase) phosphorylate multiple downstream substrates [1]. The duration and intensity of their activity is thought to control the response to growth factor signals [4]. Two distinct types of mutations have been identified in human diseases in various genes encoding components of this cascade. Miscoding oncogenic somatic mutations that cause tumorigenesis often confer enhanced and growth-factor independent, constitutive activity of the mutant protein. Examples are frequent somatic mutations of *KRAS* codons 12 and 13 [5] in various types of cancers in endodermal organs (pancreas, colon, lung, etc.), and the prevalent mutation in the kinase domain of *BRAF, BRAFV600E* [6], [7] in cancer cells of endodermal (thyroid, colon) and ectodermal (melanoma) tissues. Germ line mutations in *RAS* or *BRAF* introduce distinct amino acid changes from those found in somatic cancer cells, and they can cause a spectrum of developmental defects such as cardio-facio-cutaneous (CFC) syndrome and Noonan or Costello syndrome [8], but do not appear to be overtly oncogenic. Features of CFC include congenital heart defects, a characteristic facial appearance, gastrointestinal dysmotility, moderate-to-severe intellectual disability, and short stature [9], [10]. *BRAF* mutations have been documented in ∼75% of affected individuals while ∼25% have a mutation in *MEK1* or *MEK2* [10]. The *BRAF* mutations found in CFC can confer either weakly elevated kinase activity (mutations of Q257R, S467A, L485F, K499E) or impaired kinase activity versus wild-type *BRAF* (E501G and G596V) [11], [12].

The three functional mammalian RAF proteins (ARAF, BRAF and CRAF, the latter is also termed c-Raf-1) display redundant as well as specific functions. RAF enzymes form homo- and heterodimers [13]–[15]. It has been demonstrated that oncogenic mutant BRAF with impaired kinase activity are still able to transactivate CRAF because the mutation induced an active conformation of the enzyme under basal growth condition [14]. One important difference among RAF kinases is found in the regulation of phosphorylation sites. The serine 445 of BRAF is constitutively phosphorylated while the homologous site in CRAF must be phosphorylated for maximal activation [16]. This difference is thought to be the basis of the observation that a single point mutation at codon 600 results in constitutive BRAF activation, whereas the homologous mutation in CRAF does not [17]. LTP in the dentate gyrus of adult rats induces BRaf but not CRaf expression [18]. Lung tumour formation in mice by oncogenic KRAS requires CRaf, but not BRaf [19], [20]. Distinct biological features of Raf kinases are further suggested by the lack of compensation between Raf proteins in mice with genetic ablation of a single *Raf* gene and the different phenotypes of *Raf*-deficient mice ([21]; and see below).

Based on the neurological phenotypes reported for CFC, the generation, differentiation and functional maintenance of neurons is likely affected by mutated *BRAF*. However, little is known about the effects of these mutant proteins on cell cycle regulation of neural stem/precursor cells and neuronal differentiation. Mice with constitutive elimination of *BRaf* are embryonic lethal at midgestation [22]. In addition embryonic sensory and motor neurons of *BRaf*-deficient mice are not able to survive in the presence of neurotrophic survival factors. This effect is BRaf-specific and cannot be rescued by CRaf [23]. Global expression of constitutively active *BRAFV600E* in mouse tissues causes early embryonic lethality [24]. Conditional elimination of *BRaf* using Nestin-Cre demonstrated reduced expression of glial cell line-derived neurotrophic factor receptor RET in sensory dorsal root ganglion neurons at postnatal age and did not reveal an essential role of BRaf to relay the survival signal in neurons [25]. Furthermore, the essential role of BRaf for oligodendrocyte maturation and myelination during postnatal central nervous system development was demonstrated [26]. In addition, BRaf is involved in synaptic plasticity, because Cre-mediated conditional elimination of *BRaf* in neurons expressing calcium/calmodulin-dependent protein kinase II alpha showed impaired hippocampal learning and memory [27].

Many neurons of the adult brain are generated prenatally but in the cerebellum [28], olfactory bulb [29] and hippocampus [30], [31] neurons are also born in postnatal life. Neurogenesis persists even in adult mammals, including humans, in the subventricular zone (SVZ) and the subgranular zone (SGZ) of the dentate gyrus in the hippocampus [28], [32]–[34]. In the hippocampus, the majority of new cells migrate from the SGZ into the granule cell layer, differentiate into dentate granule neurons, and integrate into the existing neural circuitry [34]. Thus, neurogenesis is a cellular form of plasticity that accompanies the classical synaptic forms of plasticity. The question whether altered BRaf kinase levels or activity affect postnatal neuronal functions in the dentate gyrus and cerebellum has not been studied so far. Here, we analyse the effects of *BRaf* ablation in the postnatal mouse dentate gyrus and cerebellum. We observe that postnatal hippocampal dentate gyrus precursor cells show increased rates of proliferation besides impaired dendritic differentiation in both structures.

## Results

### Ablation of BRaf Impairs the Postnatal Growth of the Dentate Gyrus

**Figure 1:**
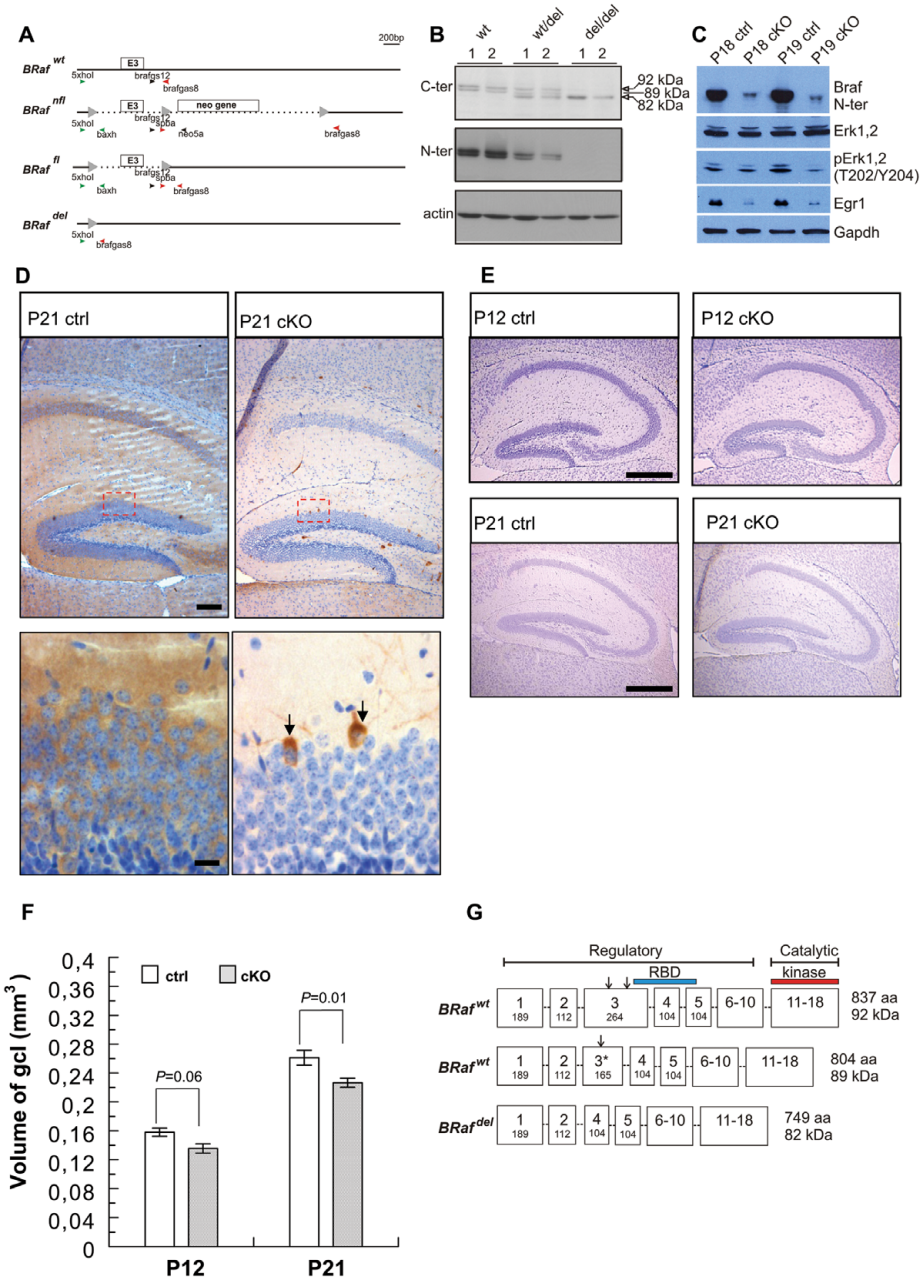
Dependence of dentate gyrus growth on BRaf. (A) Generation of conditional *BRaf* mice. In the conditional *BRaf* allele exon 3 encoding part of the Ras-binding domain is flanked by *loxP* sites (arrowheads). Deletion of the neomycin resistance gene in *BRaf ^nfl^* mice generated the *BRaf ^fl^* allele. Deletion of both exon 3 and the neomycin resistance gene generated the *BRraf ^del^* allele. Positions of primers used in PCR reactions to distinguish the different alleles are shown, for details see text. (B) Analysis of BRaf expression in embryos. Western blot analysis of BRaf expression in E10.5 embryos resulting from *BRaf ^wt/del^* intercrossing reacted with antibodies against BRaf N-terminal or C-terminal epitopes. Note that the C-terminal-specific antibody detects a ∼82 kDa BRaf band in extracts from *BRaf ^del/del^* and *BRaf ^wt/del^* embryos that is smaller than the BRaf doublet bands of ∼92 and ∼89 kDa seen in wild-type embryos. Detection of β-actin served as loading control. (C) Analysis of downstream targets of BRaf signalling. The phosphorylation levels of the kinases ERK1,2, as well as the levels of the early growth response 1 transcription factor Egr1 were significantly reduced in the hippocampus of cKO mice compared to ctrl mice whereas the expression of Erk1,2 was unaltered. The residual level of BRaf in cKO may occur from “escaper” cells. Gapdh served as loading control. (D) Analysis of BRaf expression by immunohistochemistry. Upper panels are representative sagittal sections of P21 hippocampus immunostained for BRaf with an antibody against the BRaf N-terminus. Lower panels are images taken from boxed regions in upper panels; note presence of BRaf stain in cell body of singular granule neurons (arrows) and their dendrite extending into the molecular layer that might have “escaped” Cre recombinase-mediated *BRaf* deletion in *Nestin-Cre/BRaf ^fl,fl^* mice. Scale bars; upper row, 200 µm; lower row, 25 µm. (E) Representative sagittal sections of P12 and P21 hippocampus stained with Nissl. Scale bars; 800 µm. (F) Volume of hippocampal granule cell layer (gcl) in 12 and 21 day old mice. Data are mean ±s.e.m.; P12, n = 3; P21, n = 7. (G) Exon organization and location of regulatory regions in BRaf isoforms. Boxes indicate exons with their sizes in nucleotides aligned to the regulatory, catalytic and RAS-binding domains (RBD) of BRaf protein. The vertical arrows above exon 3 indicate the positions of the 5′ end and 3′ end, respectively of an intron that has been spliced out in the small cDNA harbouring exon 3* in embryonic RNA (see Figure S2). This in-frame splicing retains the reading frame and is predicted to encode the 89 kDa BRaf isoform. The scheme is deduced from cDNA sequencing of wild-type and exon 2–4 spliced BRaf del samples (see text). The molecular masses of BRaf proteins present on the gel (Fig. 1B) are shown.

To facilitate an analysis of the role of BRaf kinase in brain development, postnatal neural stem cell proliferation and neuronal differentiation, we created a conditional allele by flanking exon 3 of the *BRaf* gene with two *loxP* sites. We targeted exon 3 because it encodes part of the Ras binding domain, which is essential for the activation of the kinase and predicted that an exon 3 deleted *BRaf* allele would be a null allele (Figure 1 A; Figure S1A). Raf kinases shuttle between closed inactive and open active conformation. The N-terminal regulatory domain maintains an inactive conformation of the kinase such that the C-terminal catalytic domain is inhibited [3], [35], [36] (Figure 1G). The RAS-binding domains (RBD), encoded by exons 3–5 and encompassing 81 amino acid residues [37] is located within the N-terminal regulatory domain (Figure 1G). Autoinhibition mediated by the regulatory domain is relieved by binding of Raf`s RBD to activated Ras. Since residues of the β-strand B2 of the RBD which are instrumental for binding to Ras/Rap are encoded in exon 3, BRaf with an exon 3 deletion would be predicted to be deficient in binding to GTP-bound Ras and shuttling Raf into the active conformation [38], [39]. All other domains in BRaf are predicted to remain intact after in-frame deletion of exon 3 (Figure 1G). We first investigated the effect of deletion of exon 3 in mice obtained from two independent targeted floxed *BRaf* ES cell clones (nfl152 and nfl156, Figure S1B, C) that were both successfully transmitted into the germ line. To obtain a line with exon 3 deletion, we crossed heterozygous female *BRaf ^wt/nfl156^* mice with male *EIIaCre* deleter mice [40]. PCR analysis of tail DNA revealed that compound offspring harbouring both the *EIIaCre* transgene and the floxed *BRaf* allele contained cells with partial or complete deletions of the *loxP* flanked regions in a mosaic fashion (not shown), in line with the known function of the *EIIaCre* strain as a general deleter. Back-crossing of these mosaic mice to wild-type mice showed that individual offspring harboured either a complete deletion of the *loxP* flanked locus (*BRaf ^wt/del^*) or a deletion removing only the neomycin resistance gene (*BRaf ^wt/fl^*; Figure 1A). The latter allele excluded any potential detrimental effect of the neo cassette in *BRaf ^wt/nfl^* mice on BRaf expression. Intercrosses of *BRaf ^wt/nfl^* or *BRaf ^wt/fl^* mice yielded homozygous *BRaf ^nfl/nfl^* and *BRaf ^fl/fl^* mice at a frequency expected for a functional allele (nfl152∶129 animals born, 31 wt, 69 wt/nfl, 29 nfl,nfl; nfl156∶72 animals born, 18 wt, 37 wt/nfl, 17 nfl,nfl; fl156∶69 animals born, 18 wt, 31 wt/fl, 20 fl,fl;). Homozygous *BRaf* ^nfl/nfl^ and *BRaf* ^fl/fl^ mice had a normal life span, were fertile and showed no macroscopic pathological phenotype in the brain. Heterozygous *BRaf ^wt/del^* mice were healthy, fertile and displayed no body weight difference compared to *BRaf ^wt/wt^* mice (Figure S1F), arguing against a dominant activity of the exon 3-deleted *BRaf^ del^* allele. In contrast, intercrosses of heterozygous *BRaf ^wt/del^* mice yielded no live born del/del offspring (Figure S1D) indicating that the deletion of exon 3 from *BRaf* had generated a non-functional BRaf expressing mouse (Figure 1G). At E10.5 already, the number of del/del embryos was lower than expected, but some of the *BRaf ^del/del^* embryos were still alive (Figure S1D), as observed previously upon constitutive ablation of *BRaf* [22]. In two litters isolated at E14.5, only one dead del/del embryo could be recovered (Figure S1D, E, G). We noted that del/del embryos as well as their placentas were smaller and retarded in their development at E10.5, compared to wt/del or wt/wt littermates (Figure S1E, and not shown). Western blot analysis from E10.5 embryo extracts with antibodies against either the N- or C-terminal domains of BRaf showed that the wild type mouse *BRaf* locus specifies the expression of two slightly different-sized isoforms of approximately 92 and 89 kDa in the embryo (Figure 1B). In the *BRaf ^wt/del^* embryo a new band was also identified, at an approximate molecular weight of 82 kDa in the immunoblot with the antibody against the C-terminus (Figure 1B). This ∼82 kDa band persisted in the del/del extracts when probed with the antibody against the C-terminus (Figure 1B). The ∼92 kDa and ∼89 kDa bands were absent in del/del embryo extracts with either of the two antibodies (Figure 1B). These protein isoforms could be due to different phosphorylation states of BRaf or to translation of alternatively spliced exons. In order to discriminate between these possibilities, we performed RT PCR on total mRNA from embryos. Using primers located in exons 1 and 4 for cDNA amplification, we observed two PCR fragments of around 420 and 320 bp in wild type embryos (Figure S2). DNA sequencing of gel-purified PCR products revealed (Figure S2) that the smaller 320 bp band corresponded to the known cDNA sequence of *BRaf* (Genebank accession NM_139294) whereas the larger 420 bp fragment had a sequence present within exon 3 that was identical to the genomic sequence (Genbank accession NC_000072). The fragment present in the wt/del embryos was a fusion of exon 2 to exon 4 (Figure S2, Figure 1G). Since the deletion of exon 3 would not change the open reading frame, the translation of a novel BRaf protein from the *BRaf ^del^* allele is plausible.

**Figure 2:**
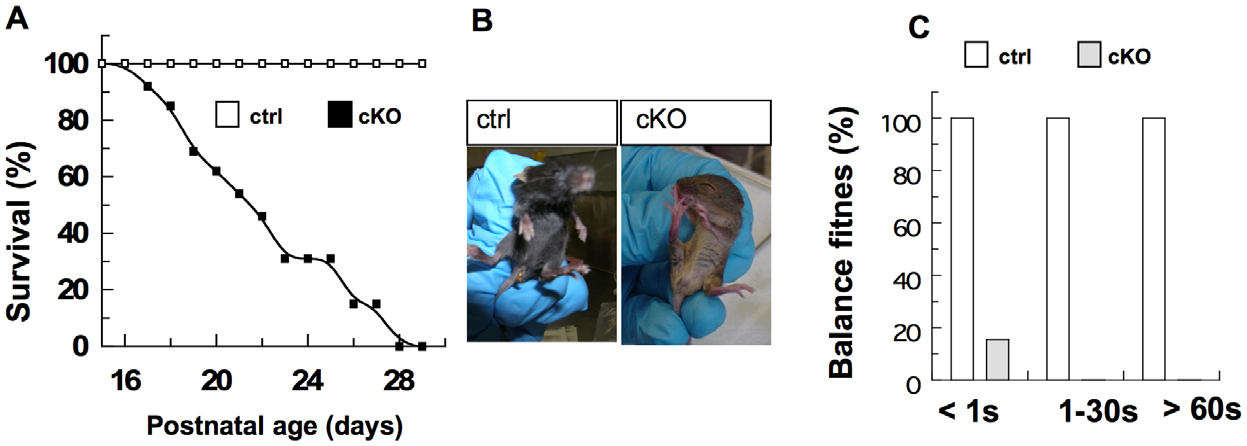
*Nestin-Cre* mediated deletion of *BRaf* causes postnatal death and abnormal behaviour. (A) Kaplan-Meier survival curves of mice with *Nestin-Cre* driven *BRaf* deletion. Mice were monitored daily. CKO, n = 13; ctrl mice, n = 10. (B) Abnormal behaviour of P21 cKO mice, indicated by autoaggression was observed in 13 out of 15 cKO mice. (C) Quantification of fraction of animals capable to balance on a small rod. CKO, n = 13; ctrl mice, n = 11.

In order to evaluate a specific role of BRaf in neural development, we crossed compound *Nestin-Cre BRaf ^wt/fl^* and *Nestin-Cre BRaf ^wt/nfl^* (ctrl) mice with *BRaf ^fl/fl^* and *BRaf ^nfl/nfl^* mice. *Nestin-Cre BRaf ^fl/fl^* and *Nestin-Cre BRaf ^nfl/nfl^* (cKO) mice were born alive at the same frequency as their ctrl littermates and all animals remained alive up to postnatal day 16 (Figure 2A). We observed no difference among the three lines of mice in terms of survival or body weights. At postnatal day 10, the body weight of cKO animals was 4.3±0.11 g, 4.0±0.15 g and 4.4±0.3 g for nfl152, nfl156 and fl animals, respectively (n = 10). Body weight in all three lines was dramatically decreased compared to control littermates (5.9±0.14 g, 6.1±0.34 g and 5.8±0.27 g, n = 14, p≤0.003). Therefore we have used both *BRaf ^fl^* and *BRaf ^nfl^* animals in our experiments. CKO mice were smaller at P5 compared to ctrl litter mates and were unable to gain weight after P10 (Figure S3A). CKO mice began to die at around P17 and none survived beyond P28 (Figure 2A). In order to analyse the level of *BRaf* elimination in hippocampi of P18/P19 mice, we performed western blot analysis of microdissected hippocampal tissue with the antibody against the N-terminal of BRaf using conditions of high signal sensitivity (see Materials and Methods) and observed that there was only a minor residual level of BRaf in cKO detectable (Figure 1C) indicating efficient gene ablation. The loss of BRaf in cKO mice could also be observed in other microdissected brain regions at P21 (Figure S3D) and in hippocampus at P6, P12 and P22 (Figure S3E). The low residual level of BRaf in cKO (Figure 1C) is likely due to a low number of BRaf-positive cells where Cre was unable to delete the *BRaf* gene as observed by immunohistochemistry (Figure 1D, Figure S4). The ∼82 kDa band was observed in ctrl and cKO mice with the antibody against the C-terminal of BRaf (not shown). In order to demonstrate that the ∼82 kDa protein is a non-functional kinase, we analysed two downstream targets of BRaf by immunblotting. We observed in brain lysates that two downstream targets of BRaf signalling, the phosphorylation levels of the kinases ERK1,2, as well as the levels of the early growth response 1 transcription factor Egr1 were significantly reduced in the hippocampus of cKO mice compared to ctrl mice (Figure 1C). This provides additional biochemical evidence that the smaller ∼82 kDa BRaf protein in our cKO mice is a non-functional kinase**.** In summary, we show that both wild-type isoforms of BRaf (∼92 kDa and ∼89 kDa) are eliminated by our Cre/loxP strategy in del/del embryos. The smaller ∼82 kDa protein expressed from the del allele represents a non-functional protein that could not act as a BRaf kinase (Figure 1G). We conclude that our targeting strategy has created a conditional *BRaf* mouse with *loxP*-flanked exon 3. The deletion of exon 3 generates a non-functional allele of *BRaf* that cannot support embryonic development and survival beyond midgestation. This phenotype is similar to that noted before in mice with constitutive ablation of *BRaf* [22] and two previously reported conditional alleles of *BRaf* where either exon 3 or exon 12 [25], [27] had been deleted.

To further analyse the *BRaf* gene deletion in the brain, we performed immunostaining of P21 brain sections. Almost all cells in the cellular hippocampal regions as well as their dendritic processes and cerebellar Purkinje cells failed to stain positive for BRaf demonstrating an efficient non-functional *BRaf* expressing mouse. Few BRaf-positive granule cells in the hippocampus and a low amount of BRaf-positive Purkinje cells in the cerebellum presumably represent cells that escaped the deletion by Cre recombinase (Figure 1D, Figure S4). To test the efficiency of Cre recombinase we performed Cre and BRaf immuno-histological staining on tissue slices. We did not expect a high amount of Cre in Purkinje cells of the cerebellum at P21, because these cells are Nestin-negative at this time point. We could not find any BRaf positive cell expressing Cre at the same time (data not shown). We therefore conclude that BRaf positive cells might result from Cre-escaping cells that have already been described earlier [41], [42].

The gross appearance of the brain and of hippocampal sections of cKO mice at around P20 did not reveal any obvious abnormality (Figure 1D, E; Figure S3F), but the weights of the brains were significantly reduced at P10 and P20 (Figure S3B). In the hippocampus, migration defects can lead to cell dispersal [43]. As BRaf has been implicated to play a role in neuronal migration in the embryonic cortex [44], we inspected hippocampal sections of cKO mice at P21 but did not observe cell dispersal in the dentate gyrus (Figure 1D, E). In order to exclude sex-related differences, quantification of the granule cell layer volume and all other subsequent experiments were performed exclusively with male littermates. A quantitative analysis of the volume of the hippocampal granule cell layer revealed that cKO mice had a reduced volume of this neuronal cell layer at P21 but not at P12 (Figure 1F). This finding indicates that BRaf has a specific role in the postnatal generation or differentiation of dentate granule neurons that are born and differentiating after P12.

### Pathophenotypes in the Cerebellum of *BRaf* Ablated Mice

**Figure 3:**
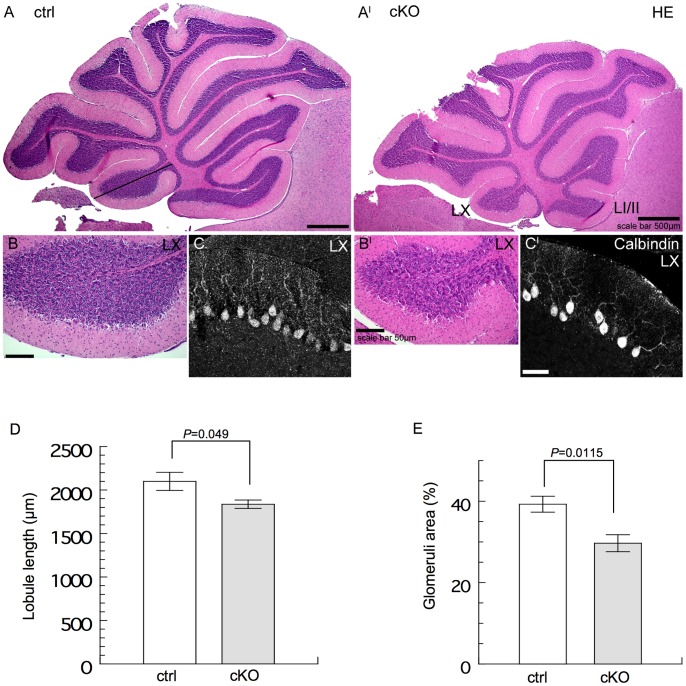
Cerebellar abnormalities caused by *Nestin-Cre* mediated deletion of *BRaf*. (A, A’) Representative sagittal sections of P21 cerebellum stained with haematoxylin and eosin (HE) are shown in the upper panel. (B,B’) HE-stained pictures display the reduced size of lobe X with disorganized glomeruli. (C, C’) Calbindin staining was used to visualize the elongated primary dendrite and the reduced and irregular arborization of Purkinje neurons in the molecular layer. Scale bars; 25 µm or as indicated. (D) Quantification of cerebellar lobule length in LV. Comparable Nissl stained slices were analysed from P21 ctrl and cKO mice. Data are mean ±s.e.m.; n = 3. (E) Glomeruli/granule cell distribution in cerebellar lobe LX. Glomeruli distribution was analysed in a defined area in three different positions of comparable slices of P21 ctrl and cKO mice. Data are mean ±s.e.m.; n = 7.

Whereas the gross appearance of the brain of cKO mice was normal (Figure S3F), their behaviour showed signs of abnormality. This included autoaggression, as evident from biting of their toes, 13 out of 15 mice showed this phenotype (Figure 2B). CKO mice could walk normally (Figure S3G). However, they showed deficiencies in their ability to walk and balance on a rod (Figure 2C). In line with this observation, several cytoarchitectonic alterations were observed in sagittal sections of the hypoplastic cerebellum of P21 cKO mice in which BRaf expression was efficiently eliminated as shown by Western blotting (Figure S3D) and immunostaining (Figure S4). The abnormalities affected all cerebellar lobes at P21 (Figure 3A, A’). Preliminary birthdating experiments suggest that altered proliferation of cerebellar progenitor cells in the second and third postnatal week may be the cause for the hypoplasticity in cKO mice. Lobuli LI/II, LIII, LVII, LIX and LX showed reduced lengths with an impairment of 30% in LI/II, 21% in LIII, 18% in LV and 23% in LX together with significant alterations in LV (n = 3, p-value 0.049) (Figure 3D). In the internal granule cell layer of lobuli LI/II, LVII and LX the boundaries of single glomeruli were less well demarcated and tended to become indistinct with fuzzy borders (Figure 3B, B’). Glomeruli were reduced in their size indicating that the synapses of the granule cells with mossy fiber and Golgi cells had not formed correctly (Figure 3B, B’). Significant alterations in granule cell/glomeruli distribution could be detected in LX (n = 7, p-value 0.0115) with an impairment of 10% of glomeruli distribution in cKO compared to ctrl mice (Figure 3E). In order to visualize Purkinje cells and their arborization, we performed calbindin staining. In the flocculonodular lobe LX of the vestibulocerebellum of cKO mice, the positions of the Purkinje cells were irregular and their total number appeared reduced (Figure 3C, C’). Notably, in cKO mice the primary dendrite of the Purkinje cells appeared elongated and the arborization in the molecular layer was reduced and irregular (Figure 3C, C’). Quantitative analysis revealed a more than 2× lengthening of primary dendrite length in cKO (LI, LIX and LX) compared to ctrl (not shown). These findings could additionally be observed in vivo using MAP2 staining of P21 mouse hippocampal slices (Figure S6).

### Ablation of *BRaf* in Neural Precursor Cells Expands the Pool of Proliferating Hippocampal Progenitor Cells in the Third Postnatal Week

**Figure 4:**
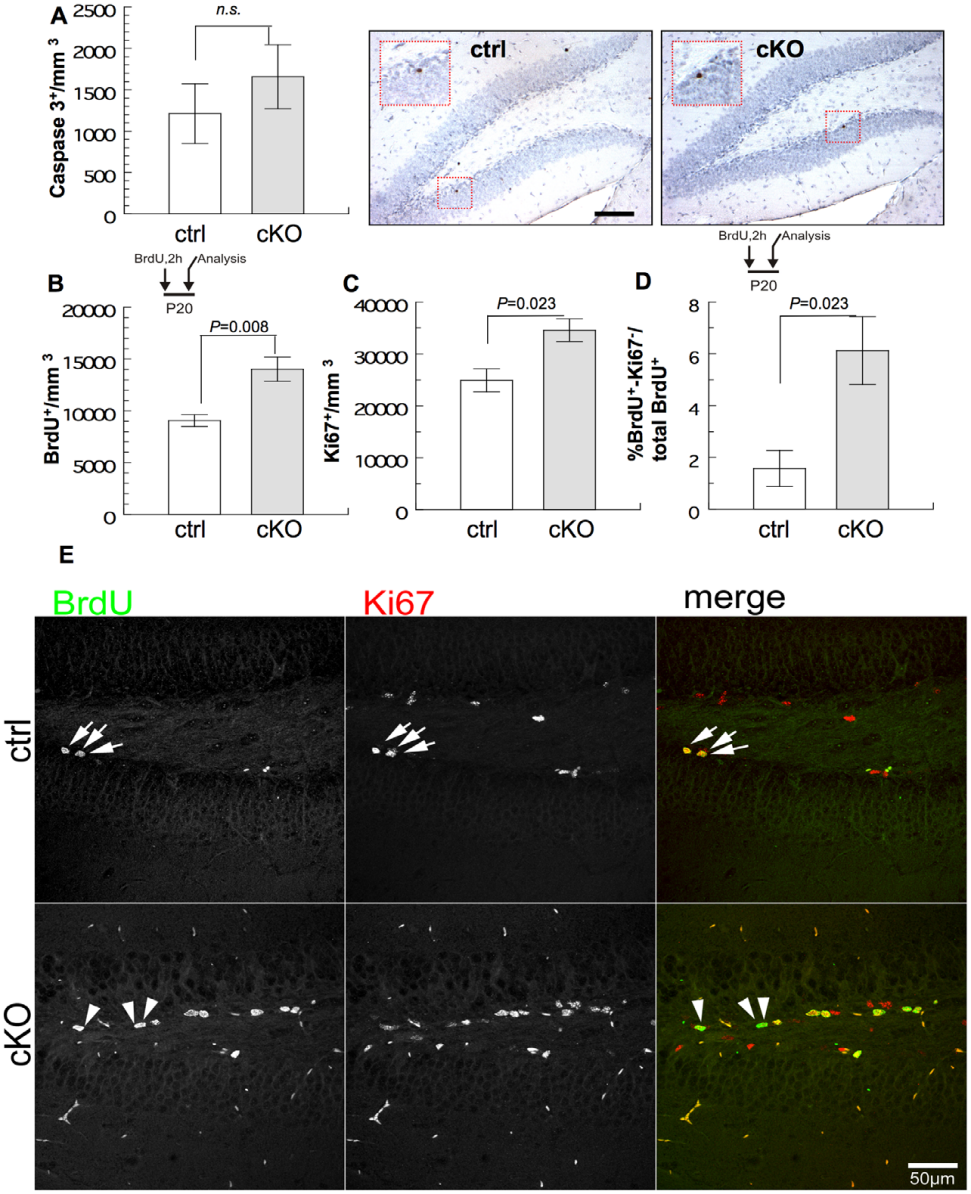
Cell cycle and cell fate analysis in postnatal hippocampus lacking BRaf. (A) Quantification of activated caspase-3-positive cells in the dentate gyrus at P24. Representative sagittal sections of the dentate gyrus of ctrl or cKO mice were stained for activated caspase-3 (brown); tissue was counterstained with Nissl. Data are mean ±s.e.m.; n = 7. Scale bar = 50 µm. (B) Quantification of BrdU-labelled cells in the dentate gyrus at P20 (2 h BrdU pulse) of ctrl or cKO mice. Data are mean ±s.e.m.; n = 4. (C) Quantification of Ki67-labelled cells in the dentate gyrus at P20 of ctrl or cKO mice. Data are mean ±s.e.m.; n = 4. (D) BrdU-positive Ki67-negative cells as a fraction of BrdU-labelled cells in the dentate gyrus at P20 of ctrl or cKO mice. Data are mean ±s.e.m.; n = 4. (E) Representative sagittal sections of the dentate gyrus of ctrl or cKO mice stained with the S-phase marker BrdU (green) and the proliferation marker Ki67 (red). Double positive cells are marked with an arrow; arrowheads depict BrdU-positive, Ki-67-negative cells. Scale bar = 50 µm.

To examine whether increased apoptosis or reduced proliferation of stem/progenitor cells could explain the reduced hippocampal granule cell volume in the absence of BRaf (Figure 1F), we first stained hippocampal sections of P24 mice for the presence of activated caspase 3. On average, one or two apoptotic cells were usually visible in a section of the dentate gyrus, and the apoptotic cells were mostly located close to the subgranular region (Figure 4A). Quantification revealed no significant increase of apoptotic cells detectable in the dentate gyrus of cKO mice, as compared to control littermates (Figure 4A). To determine whether reduced proliferation of stem/progenitor cells in the granular cell layer could be the cause of the reduced cell volume, we applied a single pulse of 5-bromo-2-deoxyuridine (BrdU) to male P20 mice and sacrificed the animals 2 h later. BrdU can be incorporated into DNA of dividing cells only during the S-phase of the cell cycle and is a useful tool for monitoring cell proliferation and birth dating [45]. Unexpectedly, BrdU immunostaining and quantification of BrdU-positive cells in the dentate gyrus of cKO mice showed an approximately 50% higher number of BrdU-positive cells as compared to ctrl mice (Figure 4B, E). As an independent measure of proliferation of neural stem cells, we stained hippocampal sections for Ki-67 protein. Since Ki-67 is expressed in all phases of the cell cycle except the resting phase [46], the fraction of Ki-67 positive cells represents the number of dividing progenitor cells. The dentate gyrus of cKO mice harboured an increased number of Ki-67 positive cells (Figure 4C, E). To address the question whether the elimination of *BRaf* in cKO mice altered the fraction of neural progenitors in the subgranular zone of the dentate gyrus that exited from the cell cycle, we applied a two-hour pulse of BrdU before tissue fixation and performed a double staining for BrdU and Ki-67. The fraction of BrdU positive cells that were negative for Ki-67 was increased in cKO mice as compared to ctrl (Figure 4D, E; BrdU-positive, Ki-67-negative cells marked with an arrowhead). We conclude that the elimination of *BRaf* mediated an increased and aberrant cell cycle exit involving loss of Ki-67 expression during the cell cycle.

### Ablation of *BRaf* in Neural Precursor Cells Impairs Neuronal Differentiation

**Figure 5:**
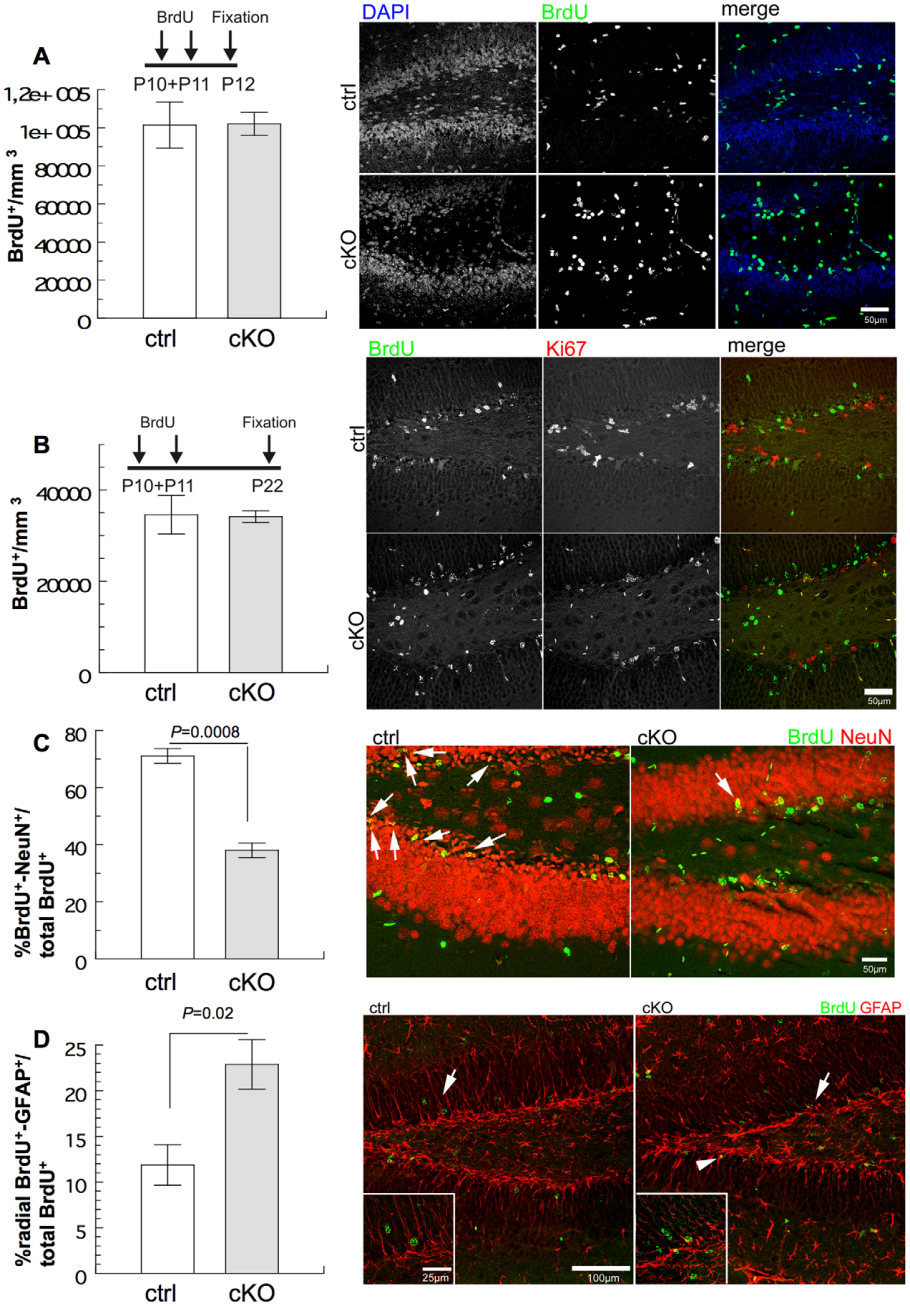
*Nestin-Cre* mediated deletion of *braf* impairs neuronal differentiation in the granular cell layer of the dentate gyrus. (A) Quantification of BrdU-labelled cells in the dentate gyrus of ctrl or cKO mice. Cells were labelled in vivo with BrdU at days P10 and P11, followed by sacrification 24 hours after the second BrdU pulse. Representative sagittal sections of the dentate gyrus stained with the proliferation marker BrdU (green). Data are mean ±s.e.m.; n = 4. Scale bar = 50 µm. (B) Quantification of BrdU-labelled cells in the dentate gyrus at P22 of ctrl or cKO mice. Neural progenitor cells were labelled in vivo with BrdU at days P10 and P11, followed by sacrification of mice at P22. Representative sagittal sections of the dentate gyrus stained with proliferation marker BrdU (green). Data are mean ±s.e.m.; n = 4. Scale bar = 50 µm. (C) Quantification of BrdU/NeuN-positive cells in the granular cell layer of the dentate gyrus of ctrl cKO mice. Neural progenitor cells were labelled in vivo with BrdU at days P10 and P11, followed by sacrification of mice at P22. Representative sagittal sections of the dentate gyrus stained with proliferation marker BrdU (green) and neuronal marker NeuN (red) 11–12 days after BrdU labelling. Double positive cells are marked with an arrow. Data are mean ±s.e.m.; n = 4. Scale bar = 50 µm. (D) Quantification of BrdU/GFAP-positive radial glia cells in the granular cell layer of the dentate gyrus of ctrl or cKO mice. Neural progenitor cells were labelled in vivo with BrdU at days P10 and P11, followed by sacrification of mice at P22. Representative sagittal sections of the dentate gyrus stained with proliferation marker BrdU (green) and neural precursor/astrocyte marker GFAP (red) 11–12 days after BrdU labelling. Expanded region is indicated by an arrow; the arrowhead depicts a double-positive cell. Data are mean ±s.e.m.; n = 4. Scale bar = 50 µm.

We were puzzled by the observation that in three week old cKO mice the volume of the dentate granule cell layer was decreased (Figure 1E), yet we observed an increased number of proliferating cells in the dentate gyrus, but no change in apoptosis (Figure 4). One potential explanation could be that the cells divide but do not differentiate into neurons which occupy a larger volume than progenitor cells with their dendritic and axonal processes. To address this point, we used birthdating experiments [45]. A cohort of mice were treated with two pulses of BrdU at days P10 and P11 and then sacrificed at P22. Using a 12 days chase period after two consecutive BrdU applications at P10 and P11, would enable us to analyse whether BrdU-labelled progenitor cells retained their radial glial GFAP molecular marker, whether they had differentiated into new NeuN-positive neurons or whether they were lost during this time interval. Notably, the number of BrdU positive cells at P12 (at the beginning of the chase period after two BrdU applications at P10 and P11) was not different in cKO mice as compared to controls (Figure 5A). This finding is in contrast to the data obtained at P20 with a two-hour BrdU pulse. The number of cells remaining BrdU positive cells at P22 after the long chase period of 12 days was reduced compared to the amount of BrdU-positive cells at the beginning of the chase period in both control and *BRaf*-deleted dentate gyrus, indicating that either the BrdU-label was diluted by cell proliferation, or by cell loss (Figure 5B). The fraction of BrdU/NeuN positive neurons was significantly reduced in cKO mice compared to controls, indicating that neuronal differentiation is impaired in the absence of BRaf (Figure 5C). In contrast, the fraction of BrdU/GFAP positive radial glia-like stem cells was approximately twofold increased in cKO mice as compared to controls (Figure 5D). The fraction of horizontal BrdU/GFAP-positive cells corresponding to differentiated astrocytes was unchanged in the absence of *BRaf*, indicating that no switch from neural to glial fate had occurred (Figure S5). Taken together, these results demonstrate an essential role of BRaf in the differentiation of precursor cells into neurons. In the absence of BRaf, the number of GFAP-positive precursor cells that were proliferating 12 days ago increased at P22 in cKO dentate gyrus.

**Figure 6:**
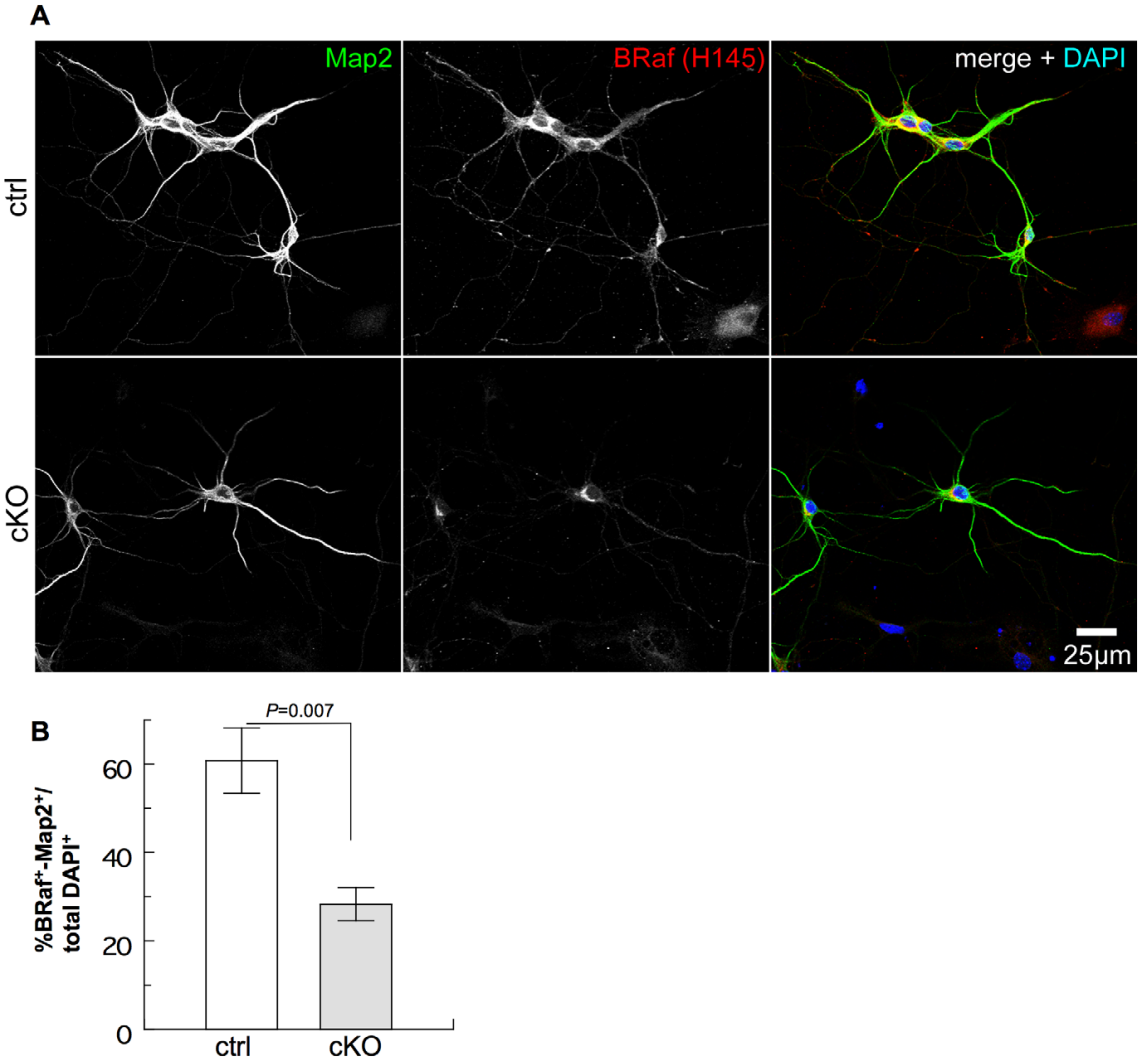
*Nestin-Cre* mediated deletion of *BRaf* impairs formation of synaptic networks of cultured hippocampal neurons. (A) Cells from the hippocampi of newborn mice were cultured for 6 days in vitro, fixed and stained for expression of BRaf and Map2. Scale bar = 25 µm**.** (B) Quantification of BRaf-positive, Map2-positive cells as a fraction of DAPI-labelled cells isolated from hippocampi at P0/P1 of ctrl or cKO mice and grown for 6 days in vitro. Data are mean ±s.e.m.; n = 5.

We used an in vitro culture system in order to investigate whether neuronal differentiation involving the growth of neurites in hippocampal neurons is impaired when *BRaf* is eliminated. Postnatal day P0/P1 hippocampi were dissected from the brain, and dissociated cell cultures were maintained in serum-free medium. After 6 days of culture, we fixed the cells and stained the samples for expression of BRaf and Map2, a marker of dendritic differentiation; nuclei were visualized by DAPI (Figure 6A, B). In hippocampal cultures from cKO mice, most of the cells had lost BRaf immunoreactivity in line with the observation in Western blots of hippocampal extracts (Figure S3D, E). These cells were unable to develop neurites as shown by the absence of Map2 staining (Figure 6A, lower panel). In control cultures however, neurons were BRaf positive and elaborated long dendrites (Figure 6A). The low number of BRaf and Map2 positive neurons in cultures from cKO mice (Figure 6A lower panel; 6B) presumably represents escapers where Cre recombinase was unable to delete the *BRaf* gene as indicated by the BRaf positive immunoreactivity in somatic areas of these neurons.

## Discussion

This study focuses on the role of the kinase BRaf in postnatal brain development. Using a conditional *loxP* recombination-site flanked allele of *BRa*f we obtained a mouse expressing non-functional BRaf that enabled the efficient deletion of functional BRaf in postnatal cerebellum, hippocampus and other brain areas of cKO compound mice. Mice expressing non-functional BRaf in neural stem cell-derived tissue survived up to four weeks. They showed alterations in the cerebellum, with reduced numbers and misplaced ectopic Purkinje cells, decreased sizes of the glomeruli in the granule cell layer and altered structure of the dendritic arborizations of the Purkinje neurons. The finding that virtually all Purkinje neurons of cKO compound animals failed to stain for BRaf expression argues against the view that a small number of “escaper” cells in which Cre recombinase did not delete exon 3 can maintain the Purkinje cell architecture. In the hippocampus, the overall cornus ammonis (CA) architecture appeared to be unchanged, but the growth of the granule cell layer in the dentate gyrus was diminished. Notably, reduced granule cell volume was obvious at P21 but normal at P12 in cKO male compound mice, compared to control littermates. In vitro cultivation of P0/P1 hippocampal cells revealed that *BRaf* deficient neurons were impaired in their differentiation into neurons lacking Map2 expressing dendrites. We propose, in analogy to the reduced arborization of the cerebellar Purkinje cells that a reduced hippocampal granule cell volume of cKO compound animals is caused also by reduced growth of their dendrites. This interpretation is supported by the absence of overt apoptosis in corresponding brain areas and by cell proliferation studies. BrdU birth-dating analysis of dentate gyrus progenitor cells at P10/P11 showed that their differentiation into NeuN-positive granule cell neurons over a 12-day period was severely reduced in mice lacking *BRaf*. Together with alterations in mouse behaviour, these observations indicate that all neuronal cellular structures develop in the absence of BRaf, but neuronal generation and maturation of dendrites are impaired in some neuronal subtypes and therefore synaptic circuits are unlikely to be fully functional. Impairment of sensory neuron differentiation has been observed before upon conditional elimination of *BRaf* [25]. Furthermore, conditional elimination of *BRaf* in neural precursors cells caused defective oligodendrocyte differentiation accompanied by dysmyelination [47].

The targeting strategy employed here is similar to the protocol chosen by Zhong and colleagues [25] in which exon 3 was also targeted for deletion by Nestin promoter driven Cre recombinase. In both strains of mice death occurs in the fourth postnatal week. Immunoblotting with a C-terminal BRaf antibody revealed the existence of a truncated, non-functional ∼82 kDa BRaf protein that most likely arises from a splice isoform from the *BRaf ^del^* allele due to splicing from exon 2 to exon 4 (see below). This splicing is plausible since there is no change of reading frame in the first four exons of *BRaf.* Nevertheless, homozygous *BRaf ^del/del^* embryos expressing the ∼82 kDa BRaf protein isoform were unable to stay alive beyond embryonic day E11. This is in accordance with observed phenotypes of mice with constitutive elimination of *BRaf* on embryonic development [22]. Notably, we demonstrate that two downstream targets of BRaf signalling, the phosphorylation levels of the kinases ERK1,2, as well as the levels of the early growth response 1 transcription factor Egr1 are significantly reduced in the hippocampus of cKO mice compared to control mice. This constitutes biochemical evidence that the smaller ∼82 kDa BRaf protein we detect in our cKO mice is a non-functional kinase.

The most prominent function of neural progenitors in the subgranular zone (SGZ) of the dentate gyrus in the hippocampus of adult mammals, including humans, is the generation of new granule cell neurons [32]–[34], [48]. It is a striking example of structural anatomical reorganization of a neuronal circuit leading to functional changes at the level of synapse formation. Notably, neurogenesis in the SGZ of the dentate gyrus is not a simple replacement event to compensate for the loss of cells, but instead leads to the addition of new glutamatergic neurons in the granule cell layer. The functional importance of adult neurogenesis has been demonstrated in mice where impaired spatial memory function was noted after elimination of the stem cells [49]. A small fraction (about 7%) of Sox2-positive neural progenitors in the subgranular zone has the potential to differentiate into astrocytes [50]. Using BrdU birth-dating in conjunction with GFAP immonostaining, we have observed an increased fraction of radial glia precursor cells. The fraction of horizontal BrdU/GFAP-positive cells corresponding to differentiated astrocytes was unchanged in the absence of BRaf indicating that the cell fate of the progenitor cell progeny did not change from neuron to glia, as has been observed for example upon transient Notch activation [51]. The use of Cre transgenic mice that are specific for neural stem cells and can be activated in adult animals by the hormone tamoxifen, such as *CreGli1-CreER^T2^* transgenic mice [52] may provide a means for a more detailed study of the role of BRaf in adult hippocampal neurogenesis. The increased number of BrdU positive cells in the subgranular zone of P20 cKO male compound mice could reflect a reactive response. Our immunostaining data indicate that BRaf is expressed in the SGZ band of the dentate gyrus, albeit at lower levels than in the differentiated granule neurons. It has been observed before that the mRNA encoding *BRaf* is expressed in the dentate gyrus and upregulated after long-term potentiation [18]. The increased fraction of BrdU positive cells that have lost Ki-67 expression is intriguing. The Ki-67 antigen is a nuclear and nucleolar protein expressed during all active phases of the cell cycle (G1, S, G2, and mitosis) and thus is a marker for proliferating precursor cells, but is absent from differentiated neurons or quiescent (G0) stem cells [46].

In our Western blot experiments of total embryonic, as well as postnatal brain extracts we observed two BRaf bands of ∼92 and ∼89 kDa, respectively. Two BRaf bands have been noted before [53]. We further observed that these two bands are absent in tissue extracts from embryos with homozygous exon 3 deletion (lanes del/del; Figure 1B). These genetic data do not support the idea that the known splicing in exons 8b/9b [54] is the basis for the isoforms observed in the immune blots but rather argue in favour of a splicing within exon 3 of the *BRaf* gene. Sequencing of RT-PCR products confirmed the existence of an intron in exon 3 that was spliced from the mRNA such that in the encoded smaller BRaf protein 33 amino acid residues would be missing. This intron/spliced-out segment (GT, AAT,GGA,–,CTT,TCA,G) starts with GT at the 5′-splice site and ends with an AG at the 3′ splice site and conforms to the canonical splicing pathway [55]. The encoded protein domain is situated N-terminal in close proximity to the RAS binding domain of RAF located in conserved region CR1 which is instrumental in recruitment of RAF to the cell membrane and the phosphorylation in the activation segment (residues threonine 599 and serine 602 in CR3 of human BRAF). Whether this alternative BRaf isoform can be activated in a distinct manner remains to be determined. Dimer formation in wild-type BRaf and transactivation of CRaf by wild-type BRaf are dependent on the interaction with activated Ras via the RBD domain [13]–[15]. CRaf/BRaf heterodimerization levels have been reported to be significantly reduced in BRaf knockdown cells [56]. Therefore, a BRaf protein lacking exon 3 would be expected to be unable to dimerize.

## Materials and Methods

### Animals

Mice were housed under barrier condition in air-filtered, temperature-controlled units with a 12 h light/dark cycle with free access to food and water. Animals were monitored for signs of distress by daily inspection. All mice were of similar genetic background (≥90% C57Bl/6, ≤10% 129Sv) and maintained as heterozygotes. Nestin-Cre mice have been described elsewhere [57] and were obtained from M. Sendtner. Compound mice were obtained by intercrossing heterozygotes. Genotype analysis of tail DNA was done by PCR at the age of three weeks. All progeny were confirmed by standard PCR-based genotyping of DNA isolated from tail biopsies or yolk sacs. Embryo staging was based on the presence of a plug and considered 0.5 days post coitus. The following primer sequences were employed: BRaf (see Figure 1A for their position): The primer pair for detection of the wild-type allele (wt) was brafgs12 (5`-TGT AGC CTC GGC TGT GGA ACT C ) and brafgas8 (5′- GAG ACC AAA CCA AGG ACC TCT G) yielding a fragment of 281 bp; the primer pair for the nfl allele was brafgs12 and neo5a (5′- ACG GAG CCG GTT GGC GCC TA) yielding a fragment of 326 bp; alternatively, the primer pair 5xhoI (5′- CCT GAA AGC TGC TAG TAG AAG AC) and baxh (5′-ACA TTG TTG ATG CTG CTC GAT CC) was used yielding a fragment of 317 bp. The primer pair for the del allele was 5xhoI and brafgas8 yielding a fragment of 421 bp. Nestin-Cre, 5′-CCG TTT GCC GGT CGT GGG and 5′-CGT ATA TCC TGG CAG CGA TC, yielding a single PCR product of ∼395 bp. All animal studies were performed in accordance with German legislation and were approved by the Bavarian State authorities for animal experimentation. All animal experiments were approved by the Committee on the Ethics of Animal Experiments of the Government of Lower Franconia (Permit Number: 55.2–2531.01–83/09). All surgery was performed under Ketanest®/Rompun® anesthesia, and all efforts were made to minimize suffering.

### Generation of “Floxed” *BRaf* Mice

The targeting construct was generated by inserting a phosphoglycerate kinase promoter-neomycin resistance cassette, flanked by two loxP sites, into the *Spe*I restriction site ∼500 bp 3′ of exon 3 in a genomic fragment of *BRaf*. A third loxP site was inserted into the *Xho*I site between exons 2 and 3, ∼300 bp 5′ of exon 3 (Figure S1A). The targeted allele was generated via homologous recombination by introducing the linearized targeting construct into mouse embryonic stem cells using standard methods. Among ∼300 ES colonies, 12 were identified as targeted clones using a PCR assay (not shown). A 5′ ∼500 bp and a 3′ ∼600 bp genomic DNA fragment (shown in Fig. S1A) were used as probes for Southern blot confirmation of ES clones harbouring the targeted allele (Figures S1B and S1C). Four targeted clones were injected into C57BL/6J blastocysts and then placed into pseudopregnant C57BL/6J females. Two clones gave germ line transmission. Chimeric mice carrying the floxed *braf* allele were crossed to C57BL/6J mice. The *BRaf ^wt/nfl^* progeny of that cross were then interbred to obtain *BRaf ^nfl/nfl^* mice from both ES clones. In addition, *BRaf ^wt/nfl^* progeny from one ES clone was crossed to mice expressing Cre recombinase under the control of the adenovirus EIIa promoter [40] to remove the neo cassette yielding *Bra ^wt/fl^* mice (lacking the neo cassette in the *BRaf* allele) and *BRaf ^wt/del^* (lacking exon-3 in the *braf* allele, Figure 1A). The PCR for the deletion of the neo cassette employed the primer pair spba (5′- TGG CAC TTA AAT ATA AGT ACT AGA TC) and brafgas8 yielding a fragment of 161 bp. Homozygous *BRaf ^nfl/nfl^* and *BRaf^fl/fl^* were born at Mendelian ratios, were viable and fertile and showed no obvious phenotype. The presence of the neo cassette in *BRaf ^fl/fl^* mice did not alter *BRaf* expression at the mRNA level, as assessed by semi-quantitative RT PCR compared to wild type controls (not shown). For conditional removal of the targeted *BRaf* gene in neural progenitors, *BRaf ^fl/fl^* mice were crossbred with *Nestin-Cre* mice [57]. Nestin-Cre mice were obtained from M. Sendtner.

### Birthdating and Histological Analysis

For cell proliferation studies we employed male littermates that were injected intraperitoneally with bromodeoxyuridine (BrdU) (50 µg/g of body weight). After appropriate labelling periods, mice were anaesthetized with Ketanest®/Rompun® and perfused transcardially with saline, followed by 4% paraformaldehyde (PFA) in PBS, pH7.4. Brains were dissected and postfixed in 4% PFA/PBS overnight at 4°C and subsequently embedded in paraffin and serially sectioned as described [44]. The left part of the brain was serially cut into a spate of ten 10 µm sagittal sections always using the first slide for Nissl staining. Quantified data were normalized to the Nissl stained DG-volume data which were measured from the beginning of the DG formation until its fusion, summarized and multiplied with slide thickness. Volume data were mentioned in mm^3^. Cerebellar lobuli length was measured from the central core axis outward including molecular layer size in comparable slices of Nissl or HE stained sections. Glomeruli/granule cell distribution in the cerebellum of Nissl stained sections was analysed using Keyence 9000 brightness quantification software in three comparable selected areas in each mice to normalize data to a defined area size (µm^2^). Glomeruli parts were shown as % of analysed area. Primary dendrite length of cerebellar Purkinje cells was analysed using Keyence 9000 measuring software. Dendrite length was quantified from the Purkinje cell body until the first arborization. Analysed data are shown in µm.

### Immunohistochemistry and Immunofluorescence Analysis

Immunohistochemical analysis was performed as described [44] **.** Antigen demasking was performed by carefully boiling the sections in a microwave oven in 10 mM citrate buffer pH6.0. Antibodies against the following proteins were used: rat anti-BrdU (Abcam ab6326, 1∶200), mouse anti-calbindin (Sigma C9848, 1∶1.000) rabbit anti-activated caspase 3 (Cell Signaling, #9661), rabbit anti-Ki67 (Thermo Scientific SP6), chicken anti-Map2 (Abcam ab5392, 1∶200), rabbit anti MAP2 (Abcam ab32454), mouse anti Cre (Abcam ab24607), rabbit anti Cre (Novagen #69050-3), mouse anti-NeuN (Millipore MAB337 or MAB337B), rabbit anti-GFAP (Dako, Z0334) and rabbit anti-BRaf (Santa Cruz, C-19, sc-166 and H-145, sc-9002). Calbindin staining was amplified by incubation with biotinylated rabbit anti mouse IgG followed by incubation with streptavidin Alexa555. BRAF (H145)-, Cre- and MAP2 staining have been performed with biotinylated anti rabbit in combination with ABC (vector) and DAB (Sigma). In all other staining procedures, appropriate species-specific Alexa488- or Cy3-labelled secondary antibodies were used for visualization with epifluorescence (Keyence Biozero 8000 equipped with a PlanFluor 20×, 0.50 NA objective (Nikon)) or confocal microscopy (Olympus FV1000 equipped with a NPLFLN 40× o NA:1.3 oil objective (Olympus)) or confocal Leica SP5 microscope equipped with a 40× 1.25–0.75 oil objective.

### Biochemistry

For Western blot analysis, tissue was lysed by mechanical homogenization in 50 mM Tris-HCl (pH 7.5), 150 mM NaCl, 2 mM EDTA, 1% (v/v) Nonidet P-40, 0.5% (w/v) sodium deoxycholate in the presence of a proteinase inhibitors (cOmplete Protease Inhibitor Cocktail, Roche) and a phosphatase inhibitor cocktail (PhosSTOP, Roche). Cell debris was removed by centrifugation at 20,000×g, and the supernatant was subjected SDS/polyacrylamide gel electrophoresis and electroblotted to nitrocellulose membranes (Whatman Protan) as described [44]. Rabbit anti-BRaf (Santa Cruz, C-19, sc-166 and H-145, sc-9002), rabbit anti Egr1 (Cell Signaling, #4153), rabbit anti phospho-ERK1,2 (Thr202/Tyr204, Cell Signaling #9101), rabbit anti ERK2 (Santa Cruz, sc-154), mouse anti GAPDH (Calbiochem CB1001), rabbit anti-β-actin antibodies (Santa Cruz, sc-1616-R)) were used. The membranes were developed by chemiluminescence detection using ECL and ECL plus (Figure 1B, Figure S3D–E) or ECL prime (Figure 1C), GE Healthcare, with a goat horseradish peroxidase-conjugated secondary antibody. The images were recorded on x-ray film.

### RNA Isolation and RT-PCR Analysis

Total RNA was extracted from embryo tissue using Trizol followed by purification with spin columns from the Pure Link RNA Mini Kit (Ambion). cDNA was synthesized with recombinant M-MuLV reverse transcriptase using 2 µg of total RNA using random hexamer primers (Fermentas). Aliquots of the reaction mixture were used for the subsequent PCR amplification. The primer sequences for BRaf amplification were 5`- GAC CCG GCC ATT CCT GAA G (sense, located in exon 1) and 5`- CTG TCT CGA ACT GTA ACA CCA CAT (antisense, located in exon 4). PCR conditions were as follows: a reaction volume of 30 µl for 5 minutes at 95°C for initial denaturation, followed by 35 cycles of 30 seconds at 95°C, 30 seconds at 60°C, 30 seconds at 72°C, and a final extension at 72°C for 10 minutes. PCR products were visualized on 2.5% agarose gels stained with ethidium bromide. Reactions without reverse transcriptase were used as a negative control. The RT-PCR for ß-actin with primers (sense, 5′-GTA TGG AAT CCT GTG GCATC; antisense, 5′ AAG CAC TTG CGG TGC ACG A) was used to check the quality of the RNA extraction and reverse transcription.

### Hippocampal Cell Culture and Staining

Hippocampi were dissected from postnatal day P0/P1 pups and treated with trypsin (15 min, 37°C, 1%, Worthington). After addition of trypsin inhibitor, 25.000 cells were cultured on 10 mm poly-L-lysine coated coverslips in 100 µl neurobasal medium containing B27 and glutamax. After 6 days culturing in vitro, cells were fixed and stained for immunofluorescence.

### Mouse Behavioural Analyses

Walking and balancing on a pencil was used to measure motor coordination; the time period spent on the rod was determined; cut-off time of this assay was one minute. Control mice could balance without problem for even longer time periods.

### Statistical Analysis

Data are presented as mean ± s.e.m. Paired t-test (two-tailed) was used to compare two groups (*P*<0.05 was considered significant). Data values in each group were assessed for normal distribution using Statsistica 8 (Statsoft) quantile plot test.

## Supporting Information

Figure S1 **Generation of the conditional** ***BRaf*** **allele.** (A) Gene targeting of the *BRaf* gene by homologous recombination in mouse embryonic stem cells was used to generate the *BRaf ^nfl^* allele. *LoxP* sequences were introduced to flank exon 3 which encodes part of the Ras-binding domain. The neomycin gene was inserted for positive selection downstream of exon 3, followed by a third *loxP* site. The position of the probes for Southern blot hybridization is shown. B, BamHI; Sp, SpeI, Xb, XbaI, Xh, Xho I. (B) Southern blot analysis using 5′ probe of five targeted ES clones, identified by PCR screening and a non-targeted clone (wt). Genomic DNA was digested with Bam HI, targeted *braf* allele is represented by the 10.3 kb band. (C) Southern blot analysis using 3′ probe of five targeted ES clones, identified by PCR screening and a non-targeted clone (wt). Genomic DNA was digested with Bam HI, targeted *braf* allele is represented by the 10.3 kb band. (D) Genotyping data from intercrosses of heterozygous *BRaf ^wt/del^* mice reveal a recessive embryonic lethal phenotype of the *BRaf ^del/del^* allele, with lethality starting at day E10.5. (E) Appearance of *BRaf ^del/del^* and control embryos. Whole-mount photographs of E10.5 embryos (left panel) and E14.5 embryos (right panel). Right embryo is *BRaf ^del/del^*; left embryo is *BRaf ^wt/del^*. (F) The *BRaf^ del^* allele does not confer any obvious dominant-negative effect on postnatal development. Body weight of P45 female *BRraf ^wt/wt^* mice compared to *BRaf ^wt/del^* littermates. Data are mean s.e.m.; n = 5 for *BRaf ^wt/wt^*, n = 9 for *BRaf ^wt/del^* group. (G) PCR-based genotyping to distinguish *BRaf ^wt/del^* (lanes 1 and 2), *BRaf ^del/del^* (lane 3) and *BRaf ^wt/wt^* (lane 4) genotypes.
(TIF)

Figure S2 **Expression of distinct transcripts of BRaf in embryos.** RT-PCR amplifications of *BRaf* and *β–actin* (to check the quality of RNA extraction and reverse transcription) using RNA isolated from E10.5 embryos. Scheme depicting the *BRaf* gene; the primers for RT PCR and the expected sizes of the PCR products are given. The vertical arrows above exon 3 indicate the positions of the 5′ end and 3′ end, respectively of an intron that has been spliced out in the small cDNA. Two different transcripts, originating from alternative splicing in exon 3, are expressed in the embryo. In *BRaf ^wt/del^* embryos, an internally truncated transcript lacking exon 3 and originating from the fusion of exon 2 to exon 4 is expressed. Sequences of the gel-purified fragments are given.
(TIF)

Figure S3 **Abnormalities caused by** ***Nestin-Cre*** **mediated deletion of** ***BRaf.*** (A) Lack of animal growth in cKO mice after postnatal day 10 (Points, mean, bars, ± s.e.m., ***, *P*<0.0001, n = 5 mice for each time point). (B) Brain weight of ctrl and cKO mice at postnatal days 10 or 20 (Points, mean, bars, ± s.e.m., n = 5 mice for each time point). (C) Brain weight in % of body weight in ctrl and cKO mice at postnatal day P10 and P20. (D) Western blot analysis of BRaf expression in P21 dissected brain regions (hp, hippocampus; pc, prefrontal cortex; cb, cerebellum; bo, olfactory bulb) of ctrl and cKO mice. Detection of β-actin served as loading control. (E) Analysis of BRaf expression in the postnatal hippocampus after *Nestin-Cre* mediated *BRaf* ablation. Western blot analysis with the antibody against the N-terminal of BRaf in lysates from micro-dissected hippocampi of P6, P12 and P22 ctrl (b-raf +,fl) or cko (b-raf fl,fl) mice. Detection of β-actin served as loading control. (F) Macroscopic appearance of 20 day old brains of ctrl or cKO mice. (G) Walking traces of 20 days old ctrl or cKO mice.
(TIF)

Figure S4 **Analysis of** ***Nestin-Cre*** **mediated deletion of** ***BRaf*** **in the postnatal cerebellum by immunohistochemistry.** Representative sagittal sections of P21 cerebellum of cKO animals (middle panels) immunostained for BRaf with an antibody against the BRaf N-terminus demonstrate widespread absence of BRaf immunoreactivity, as compared to sections from ctrl mice (upper panels). BRaf elimination is demonstrated in the lobulus X. Note presence of BRaf stain in cell body of singular Purkinje neurons that might have “escaped” Cre recombinase-mediated *BRaf* deletion in cKO mice. Control slices were incubated in blocking solution containing secondary antibody related serum in the absence of primary antibody dilution (lower panels) to visualize unspecific background staining.
(TIF)

Figure S5 **Lack of increased astrocytic differentiation in the dentate gyrus.** Quantifications of BrdU/GFAP-positive astrocytes (horizontal glia cells) in the granular cell layer of the dentate gyrus of ctrl or cKO mice. Neural progenitor cells were labelled in vivo with BrdU at days P10 and P11, followed by sacrification of mice at P22 and stained with proliferation marker BrdU and the astrocyte marker GFAP. Data are mean ±s.e.m.; n = 3.
(TIF)

Figure S6 **Dendritic morphology of hippocampal neurons is affected by** ***Nestin-Cre*** **mediated deletion of** ***BRaf*** **.** Sagittal paraffin sections of P21 mice were labelled with anti-MAP2 antibodies to visualize the dendritic morphology of neurons in the hippocampal region. Representative images of dendritic processes of pyramidal neurons in the subiculum (A, A’) and in the CA1 region (B, B’). Wild type animals (ctrl, left panel) are compared with cKO (right panel). Arrowheads indicate regions where the lengthening of the primary dendrite morphology of cKO is altered compared to ctrl (arrows). (C, C’) Negative control slides of the hippocampal CA1 region are labelled with secondary antibody only.
(TIF)
