# Supplementary figures and images for: Ablation of BRaf Impairs Neuronal Differentiation in the Postnatal Hippocampus and Cerebellum
Source: PLoS One. 2013 Mar 7;8(3):e58259. doi: 10.1371/journal.pone.0058259 (PMC3591433; doi:10.1371/journal.pone.0058259)

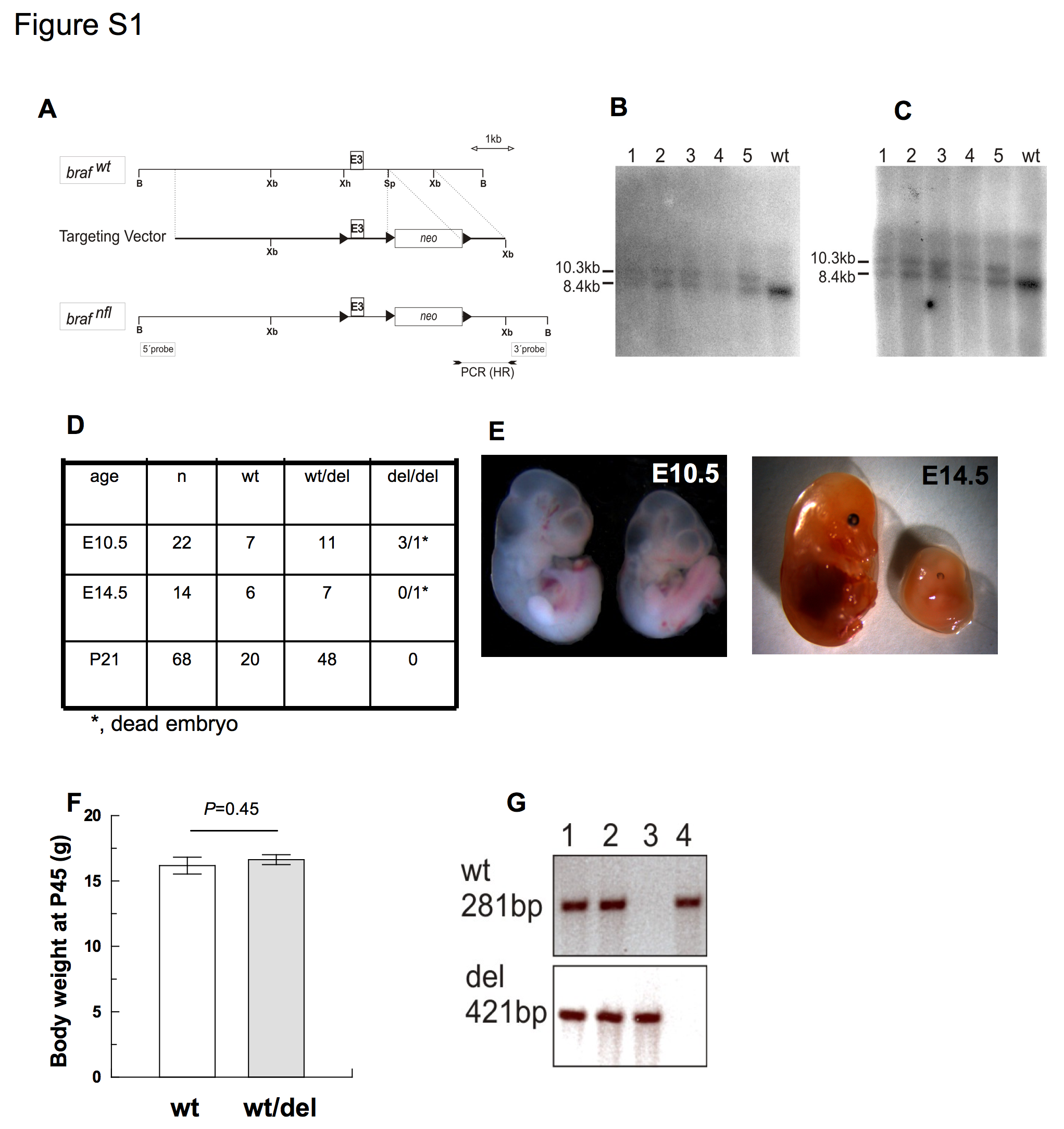

Supplement: Figure S1 — Generation of the conditional BRaf allele. (A) Gene targeting of the BRaf gene by homologous recombination in mouse embryonic stem cells was used to generate the BRaf nfl allele. LoxP sequences were introduced to flank exon 3 which encodes part of the Ras-binding domain. The neomycin gene was inserted for positive selection downstream of exon 3, followed by a third loxP site. The position of the probes for Southern blot hybridization is shown. B, BamHI; Sp, SpeI, Xb, XbaI, Xh, Xho I. (B) Southern blot analysis using 5′ probe of five targeted ES clones, identified by PCR screening and a non-targeted clone (wt). Genomic DNA was digested with Bam HI, targeted braf allele is represented by the 10.3 kb band. (C) Southern blot analysis using 3′ probe of five targeted ES clones, identified by PCR screening and a non-targeted clone (wt). Genomic DNA was digested with Bam HI, targeted braf allele is represented by the 10.3 kb band. (D) Genotyping data from intercrosses of heterozygous BRaf wt/del mice reveal a recessive embryonic lethal phenotype of the BRaf del/del allele, with lethality starting at day E10.5. (E) Appearance of BRaf del/del and control embryos. Whole-mount photographs of E10.5 embryos (left panel) and E14.5 embryos (right panel). Right embryo is BRaf del/del; left embryo is BRaf wt/del. (F) The BRaf del allele does not confer any obvious dominant-negative effect on postnatal development. Body weight of P45 female BRraf wt/wt mice compared to BRaf wt/del littermates. Data are mean s.e.m.; n = 5 for BRaf wt/wt, n = 9 for BRaf wt/del group. (G) PCR-based genotyping to distinguish BRaf wt/del (lanes 1 and 2), BRaf del/del (lane 3) and BRaf wt/wt (lane 4) genotypes. (TIF) [file pone.0058259.s001.tif]

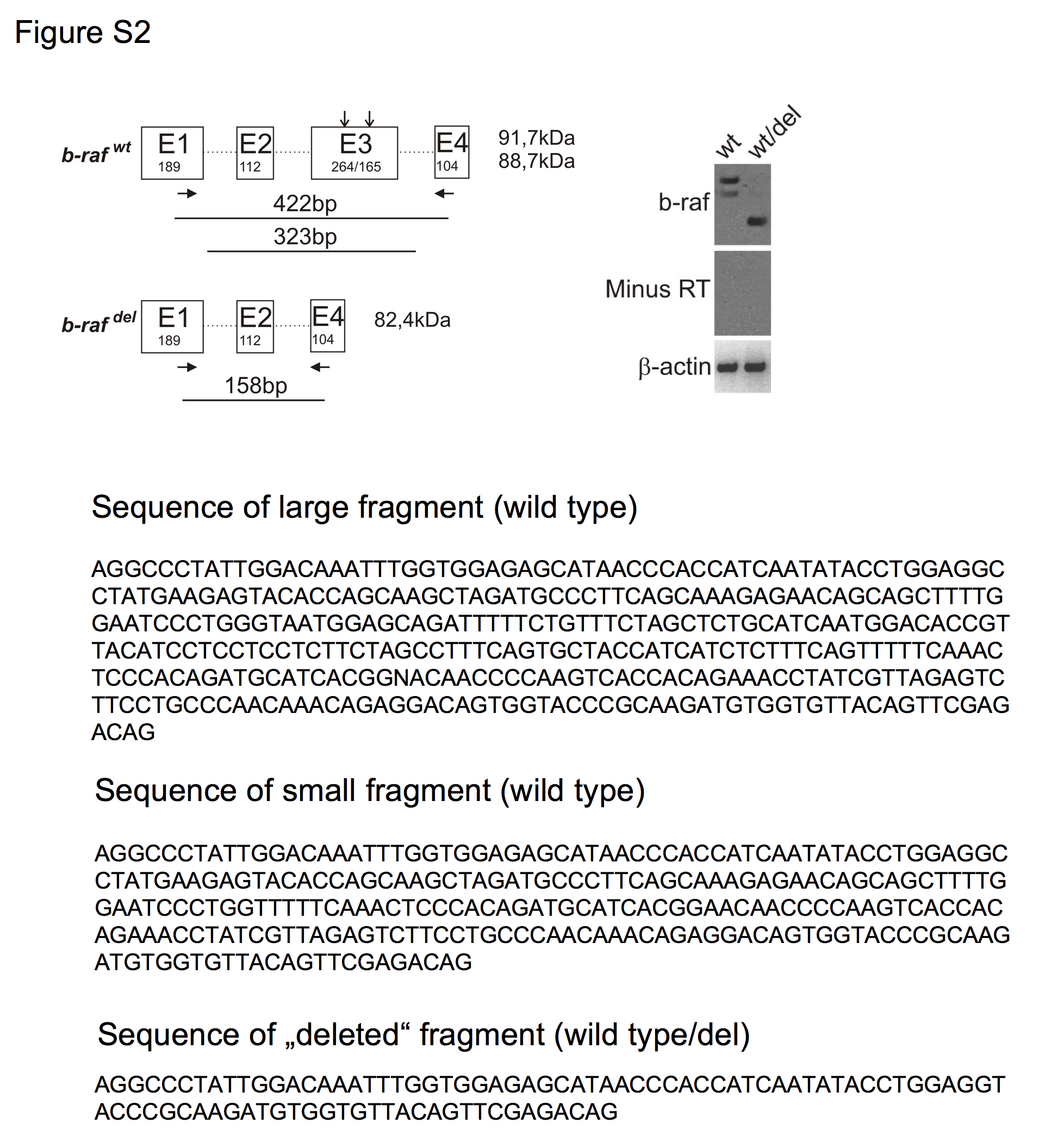

Supplement: Figure S2 — Expression of distinct transcripts of BRaf in embryos. RT-PCR amplifications of BRaf and β–actin (to check the quality of RNA extraction and reverse transcription) using RNA isolated from E10.5 embryos. Scheme depicting the BRaf gene; the primers for RT PCR and the expected sizes of the PCR products are given. The vertical arrows above exon 3 indicate the positions of the 5′ end and 3′ end, respectively of an intron that has been spliced out in the small cDNA. Two different transcripts, originating from alternative splicing in exon 3, are expressed in the embryo. In BRaf wt/del embryos, an internally truncated transcript lacking exon 3 and originating from the fusion of exon 2 to exon 4 is expressed. Sequences of the gel-purified fragments are given. (TIF) [file pone.0058259.s002.tif]

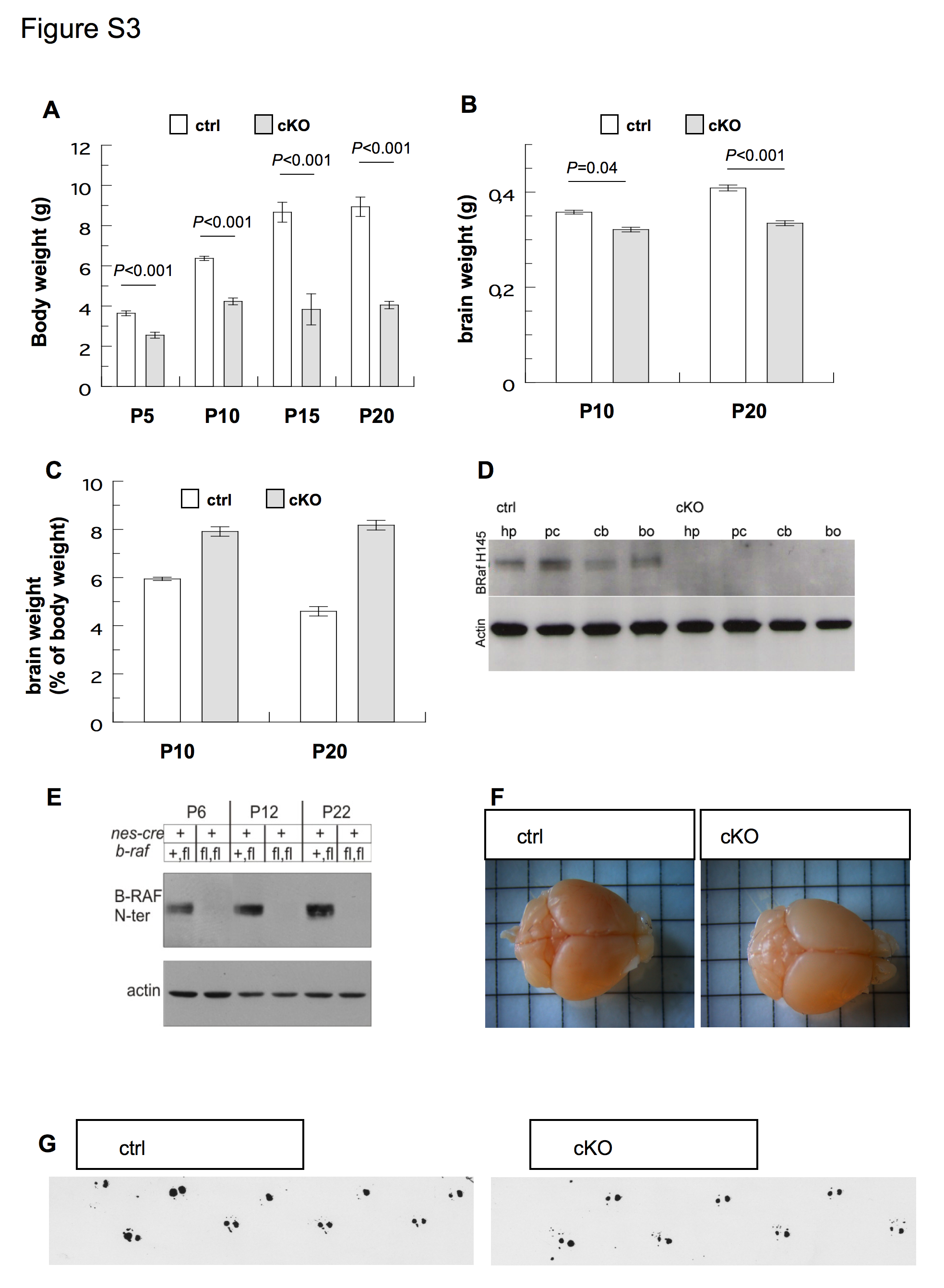

Supplement: Figure S3 — Abnormalities caused by Nestin-Cre mediated deletion of BRaf. (A) Lack of animal growth in cKO mice after postnatal day 10 (Points, mean, bars, ± s.e.m., ***, P<0.0001, n = 5 mice for each time point). (B) Brain weight of ctrl and cKO mice at postnatal days 10 or 20 (Points, mean, bars, ± s.e.m., n = 5 mice for each time point). (C) Brain weight in % of body weight in ctrl and cKO mice at postnatal day P10 and P20. (D) Western blot analysis of BRaf expression in P21 dissected brain regions (hp, hippocampus; pc, prefrontal cortex; cb, cerebellum; bo, olfactory bulb) of ctrl and cKO mice. Detection of β-actin served as loading control. (E) Analysis of BRaf expression in the postnatal hippocampus after Nestin-Cre mediated BRaf ablation. Western blot analysis with the antibody against the N-terminal of BRaf in lysates from micro-dissected hippocampi of P6, P12 and P22 ctrl (b-raf +,fl) or cko (b-raf fl,fl) mice. Detection of β-actin served as loading control. (F) Macroscopic appearance of 20 day old brains of ctrl or cKO mice. (G) Walking traces of 20 days old ctrl or cKO mice. (TIF) [file pone.0058259.s003.tif]

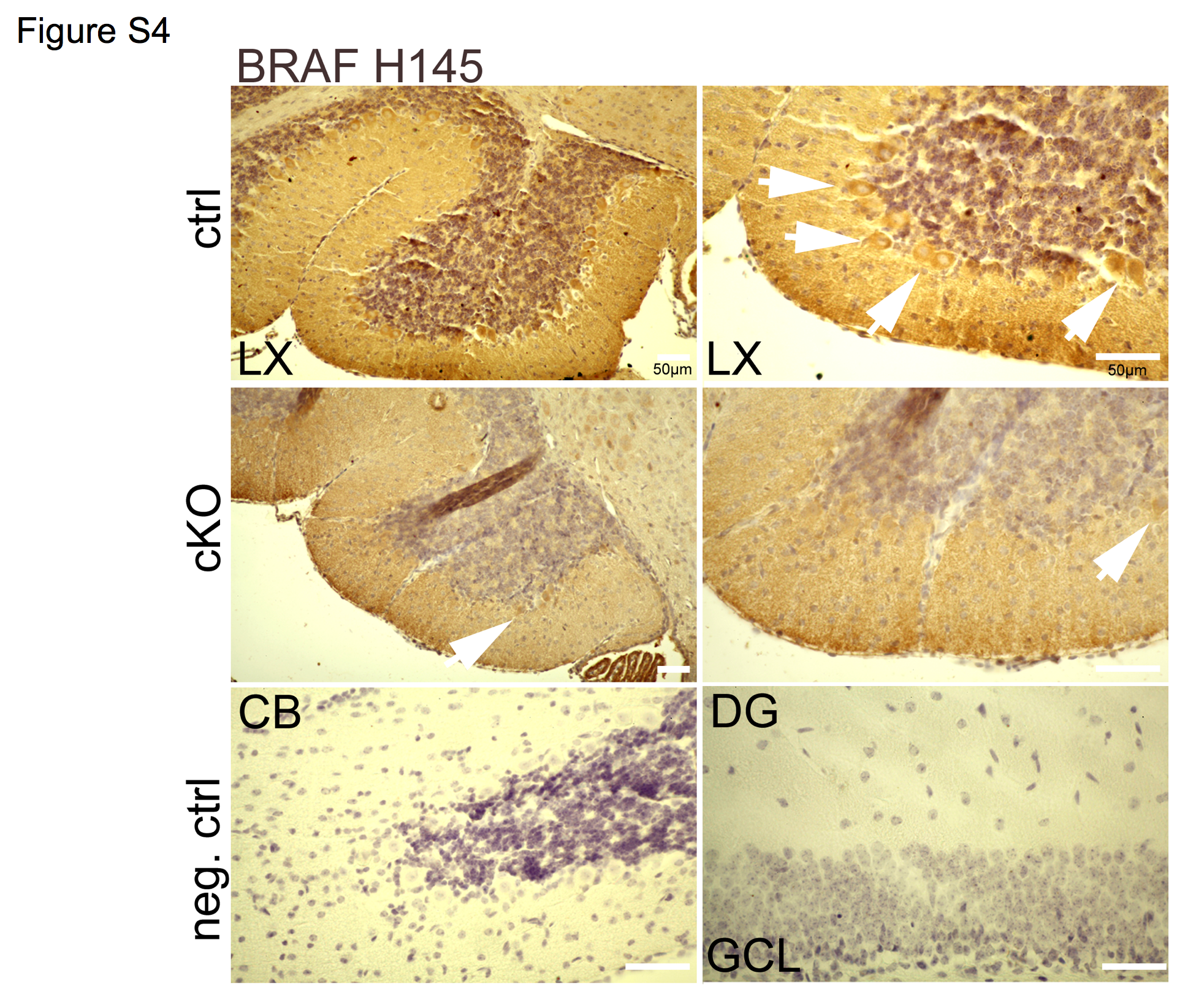

Supplement: Figure S4 — Analysis of Nestin-Cre mediated deletion of BRaf in the postnatal cerebellum by immunohistochemistry. Representative sagittal sections of P21 cerebellum of cKO animals (middle panels) immunostained for BRaf with an antibody against the BRaf N-terminus demonstrate widespread absence of BRaf immunoreactivity, as compared to sections from ctrl mice (upper panels). BRaf elimination is demonstrated in the lobulus X. Note presence of BRaf stain in cell body of singular Purkinje neurons that might have “escaped” Cre recombinase-mediated BRaf deletion in cKO mice. Control slices were incubated in blocking solution containing secondary antibody related serum in the absence of primary antibody dilution (lower panels) to visualize unspecific background staining. (TIF) [file pone.0058259.s004.tif]

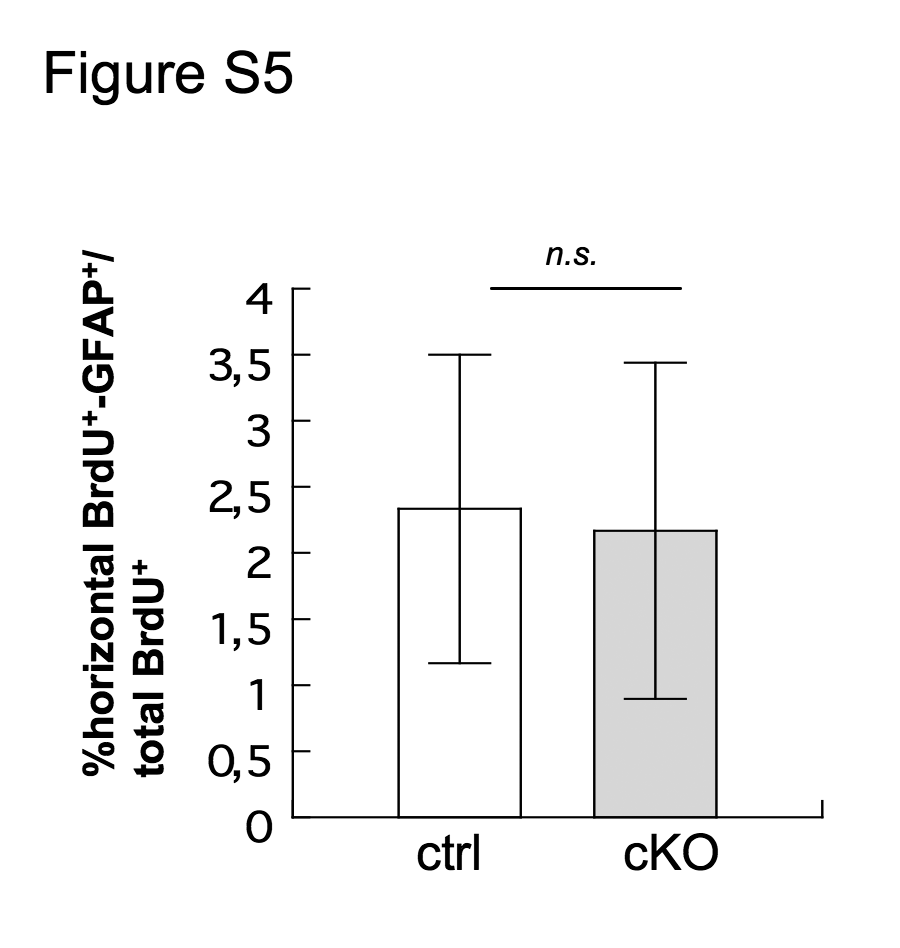

Supplement: Figure S5 — Lack of increased astrocytic differentiation in the dentate gyrus. Quantifications of BrdU/GFAP-positive astrocytes (horizontal glia cells) in the granular cell layer of the dentate gyrus of ctrl or cKO mice. Neural progenitor cells were labelled in vivo with BrdU at days P10 and P11, followed by sacrification of mice at P22 and stained with proliferation marker BrdU and the astrocyte marker GFAP. Data are mean ±s.e.m.; n = 3. (TIF) [file pone.0058259.s005.tif]

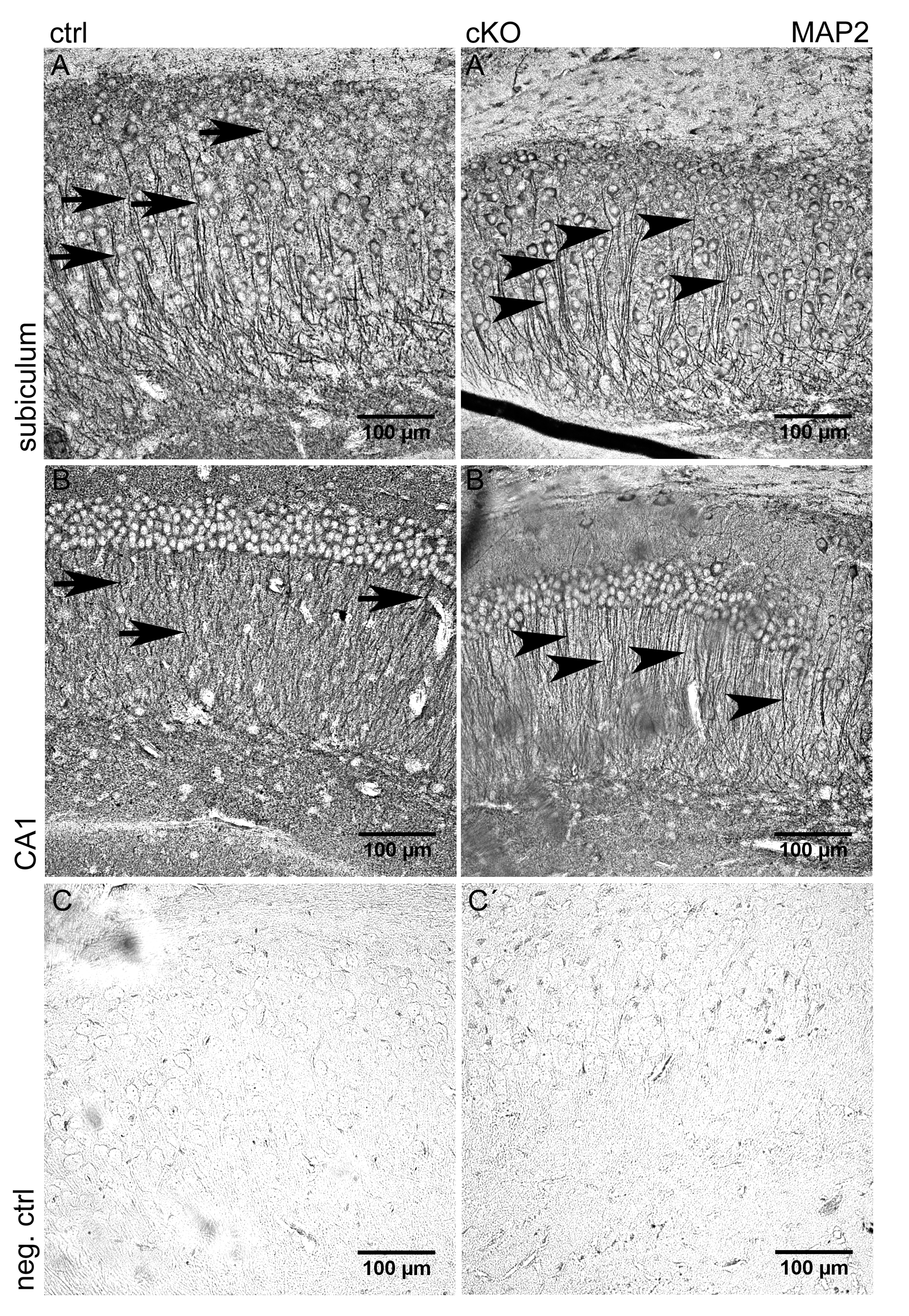

Supplement: Figure S6 — Dendritic morphology of hippocampal neurons is affected by Nestin-Cre mediated deletion of BRaf . Sagittal paraffin sections of P21 mice were labelled with anti-MAP2 antibodies to visualize the dendritic morphology of neurons in the hippocampal region. Representative images of dendritic processes of pyramidal neurons in the subiculum (A, A’) and in the CA1 region (B, B’). Wild type animals (ctrl, left panel) are compared with cKO (right panel). Arrowheads indicate regions where the lengthening of the primary dendrite morphology of cKO is altered compared to ctrl (arrows). (C, C’) Negative control slides of the hippocampal CA1 region are labelled with secondary antibody only. (TIF) [file pone.0058259.s006.tif]
